# Minimal intervention dentistry for managing carious lesions into dentine in primary teeth: an umbrella review

**DOI:** 10.1007/s40368-021-00675-6

**Published:** 2021-11-16

**Authors:** A. BaniHani, R. M. Santamaría, S. Hu, M. Maden, S. Albadri

**Affiliations:** 1 Department of Paediatric Dentistry, School of Dentistry, University of Leeds, Leeds, UK; 2 Department of Preventive and Paediatric Dentistry, University of Greifswald, Greifswald, Germany; 3 Faculty of Dentistry, National University of Singapore, Singapore, Singapore; 4 Liverpool Reviews & Implementation Group, University of Liverpool, Liverpool, UK; 5 School of Dentistry, University of Liverpool, Pembroke place, Liverpool, L3 5PS UK

**Keywords:** Minimal intervention dentistry, Dentine caries, Primary teeth, Non-operative, Selective caries removal, Sealing dental caries

## Abstract

**Purpose:**

This umbrella review systematically appraised published systematic reviews on Minimal Intervention Dentistry interventions carried out to manage dentine carious primary teeth to determine how best to translate the available evidence into practice, and to provide recommendations for what requires further research.

**Method:**

An experienced information specialist searched MEDLINE, Embase, Cochrane Database of Systematic Reviews, Epistemonikos, Joanna Briggs Institute Database of Systematic Reviews and Implementation Reports, and the NIHR Journals Library. In addition, the PROSPERO database was searched to identify forthcoming systematic reviews. Searches were built around the following four concepts: primary teeth AND caries/carious lesion AND Minimal Intervention Dentistry AND systematic review/meta-analysis. Searches were restricted to English language, systematic reviews with/without meta-analyses published between January 2000 and August 2020. Two reviewers independently screened all titles and abstracts. Interventions included involved no dentine carious tissue removal (fissure sealants, resin infiltration, topical application of 38% Silver Diamine Fluoride, and Hall Technique), non-restorative caries control, and selective removal of carious tissue involving both stepwise excavation and atraumatic restorative treatment. Systematic reviews were selected, data extracted, and risk of bias assessed using ROBIS by two independent reviewers. Studies overlap was calculated using corrected covered area.

**Results:**

Eighteen systematic reviews were included in total; 8 assessed the caries arresting effects of 38% Silver Demine Fluoride (SDF), 1 on the Hall Technique (HT), 1 on selective removal of carious tissue, and eight investigated interventions using atraumatic restorative treatment (ART). Included systematic reviews were published between 2006 and 2020, covering a defined time frame of included randomised controlled trials ranging from 1969 to 2018. Systematic reviews assessed the sealing efficacy of fissure sealants and resin infiltration in carious primary teeth were excluded due to pooled data reporting on caries arrest in both enamel and outer third of dentine with the majority of these carious lesions being limited to enamel. Therefore, fissure sealants and resin infiltration are not recommended for the management of dentinal caries lesions in primary teeth. Topical application of 38% SDF showed a significant caries arrest effect in primary teeth (*p* < 0.05), and its success rate in arresting dental caries increased when it was applied twice (range between 53 and 91%) rather than once a year (range between 31 and 79%). Data on HT were limited and revealed that preformed metal crowns placed using the HT were likely to reduce discomfort at time of treatment, the risk of major failure (pulp treatment or extraction needed) and pain compared to conventional restorations. Selective removal of carious tissue particularly in deep carious lesions has significantly reduced the risk of pulp exposure (77% and 69% risk reduction with one-step selective caries removal and stepwise excavation, respectively). ART showed higher success rate when placed in single surface compared to multi-surface cavities (86% and 48.7–88%, respectively, over 3 years follow-up).

**Conclusion:**

Minimal Intervention Dentistry techniques, namely 38% SDF, HT, selective removal of carious tissue, and ART for single surface cavity, appear to be effective in arresting the progress of dentinal caries in primary teeth when compared to no treatment, or conventional restorations. There is clear need to increase the emphasis on considering these techniques for managing carious primary teeth as a mainstream option rather than a compromise option in circumstances where the conventional approach is not possible due to cooperation or cost.

**Supplementary Information:**

The online version contains supplementary material available at 10.1007/s40368-021-00675-6.

## Introduction

Dental caries is well recognised as a controllable chronic disease that can be identified, diagnosed, and managed using biological approaches. The concept of MID for managing carious lesions has developed based on biological concepts and evidence-based outcomes of novel and existing caries control interventions which focuses on detecting carious lesions as early as possible, remineralising enamel and dentine using optimal caries control measures, and on other occasions, the use of minimal invasive operative interventions, and the concept of repair rather than replacement of restorations to arrest the progression of carious lesions (Ericson et al. 2003; Frencken et al. 2012; Dorri et al. 2015; Schwendicke et al. 2016). These concepts cover a wide number of procedures with the aim to manage carious lesions preserving as much of the tooth structure as possible.

The demineralisation process leading to dental caries can be controlled generally by reducing the intake and frequency of sugar as well as removing the dental biofilm by tooth-brushing and using a fluoride-containing toothpaste (Kidd 2011; Kidd and Fejerskov 2013). Methods to control enamel carious lesions include the use of additional fluoride (e.g. gel, varnish), pits and fissures sealants and resin infiltration. With respect to carious lesions into dentine, these measures are often no longer sufficient, and further minimally invasive (operative) interventions should be considered. Specifically for primary teeth, minimal intervention caries control strategies include a wide range of approaches, including those where carious tissue removal is not involved, such as non-restorative cavity control (NRCC) (Gruythuysen et al. 2011; Santamaria et al. 2018), sealing the carious lesion with fissure sealants (FS) and resin infiltration (Borges et al. 2012; Hesse et al. 2014; Splieth et al. 2020b; Paris et al. 2020), topical application of silver diamine fluoride (38% SDF mainly) (Chibinski et al. 2017; Richards 2017), and the Hall Technique (HT) (Innes et al. 2017; BaniHani et al. 2018; Santamaria and Innes 2018).

On a wider scope, management techniques include those in which dentine carious tissue is selectively removed to soft or firm dentine at one visit including atraumatic restorative treatment (ART) or the stepwise removal, which involves two-step carious tissue removal, both of them to avoid pulp exposure (Ricketts et al. 2013; Bjorndal 2018).

Previously, a surgical approach using a conventional rotary carious tissue removal method was used for tooth and cavity preparation focused on preparing appropriate space to place particular restorative materials. When managing carious primary teeth, the surgical method is less preferred as it removes extensive tooth structure where the tooth morphology already presents the challenges of thin enamel and dentine and relatively large pulp chambers. Furthermore, the majority of the techniques using surgical approach require the use of local anaesthesia, rubber dam and produces noise, and consequently discomfort, and fear among children in particular (Frencken et al. 2012).

In the last few decades, there has been a debate among researchers and clinicians about the advantages and disadvantages of MID over the surgical conventional approach for treating asymptomatic primary carious teeth, and the question of whether MID methods should be considered as standard techniques for management of these teeth. Nonetheless, the decision around when to use which management method should follow more recent biological evidence-based caries management concepts, which emphasises on preserving as much tooth structure as feasible and maintaining teeth functional for as long as possible, in case of primary teeth until these exfoliate naturally. In addition, caries management approaches in paediatric dentistry should be cost-effective as well as acceptable to carers and patients while causing the least possible discomfort to the child. There is evidence that MID methods used for children as caries management option are cost-effective (BaniHani et al. 2019) and have positive patients reported outcomes in terms of child’s pain perception, and technique acceptability by children and carers (Crystal et al. 2017; BaniHani et al. 2019; El-Yousfi et al. 2020; Santamaría et al. 2020).

The number of clinical studies and reviews evaluating the effectiveness of MID methods for caries management in children has significantly increased in recent years. Many of them have focused on the evaluation of success and failure of the overall treatment of specific material/treatment methods rather than the overall concept of MID. Using an umbrella review design, this study aimed to obtain a comprehensive overview of published evidence on MID interventions carried out to manage dentinal caries in primary teeth to determine how best to translate the available evidence into practice, and to provide recommendations for what requires further research.

## Methods

The aim of this umbrella review was to appraise and summarise the available evidence on the outcomes of MID interventions in the management of dentine caries, ICDAS 4 and 5, in primary teeth. This study followed the Cochrane methodology (Version 6) (Higgins et al. 2020) and the research topic was registered in PROSPERO No. (CRD42020202434).

This umbrella review asked the following PICO questions:

1. Do patients with dentine carious lesions, ICDAS 4 and 5, in primary teeth that are managed with different types of MID compared with conventional restoration approach, placebo and no treatment have different outcomes, in terms of treatment success and failure?
2. Do the following factors (presence of preoperative radiograph, depth of the dentine carious lesion, surface(s) affected (single-or multi-surface lesions), extent of carious removal, type of tooth (incisor/molar), material used for restoration, and method of caries removal significantly impact the clinical effectiveness (clinical outcomes) of MID?

### Inclusion criteria

- Participants:
  Children in their primary dentition with an untreated carious lesion(s) extending into dentine, ICDAS 4 and 5 detected clinically and/or radiographically, in primary teeth that required intervention to limit caries progression using MID without the use of local anaesthetic. Only teeth without pre-existing restorations will be considered to exclude the possibility of the dental pulp being compromised by previous treatment.
- Intervention:
  Recent consensus statements established recommendations on how and when to intervene in when managing carious lesions based on the concept of MID to assist clinical decision-making (Schwendicke et al. 2019). It was agreed that lesion activity, cavitation and cleansability should be considered as the main factors to decide on the management options to be used. Considering this, inactive carious lesions do not frequently involve any treatment in terms of lesion control, however, active carious lesions do. In terms of lesions cavitation, non-cavitated carious lesions and also cavitated carious lesions, which are cleansable should be managed non-or micro-invasively. Cavitated carious lesions, which are not cleansable usually require restorative management. Additional restorative treatment might be indicated in cases when form, function and aesthetics of the tooth needed to be restored.
  In this Umbrella review, the following interventions were included:
  o No dentine carious tissue removal: this included fissure sealants, resin infiltration, topical application of 38% SDF, and HT. Resin infiltration involves the use of a resin to seal carious lesions, preventing acid penetration and stop progression of caries. Whereas SDF (38%) is a topical colourless solution of silver, and fluoride (44,800 ppm) aims to arrest carious lesions. In HT, a preformed metal crown (PMC) is cemented over the primary molar to seal dentine carious lesions.
  o NRCC: in this treatment group, cavitated dentine carious lesions are transformed to cleansable forms that can be cleaned by the patient or carer with a toothbrush using fluoridated toothpaste.
  o Selective removal of carious tissue including both to soft and firm dentine: this included selective removal of carious tissue until either soft or firm dentine is reached to avoid pulp exposure. Periphery of the cavity is cleaned to hard (sound) dentine. This intervention arm also included stepwise removal and ART.
    Stepwise removal is a technique that involves 2-step caries removal, where selective removal to soft dentine pulpally is conducted in the first step and the cavity is provisionally restored, followed 6–12 months later by selective removal to firm dentine pulpally and placement of definite restoration. In ART, carious tissue is removed pulpally using hand instruments only and cavity is restored with high viscosity glass ionomer cement (HVGIC).
- Comparator(s)/control:
  Conventional restoration approach including non-selective caries removal to hard dentine (formally known as complete caries removal), vital pulp therapy, placebo and no treatment.

### Treatment outcomes

The main outcome of this umbrella review was “successful” measured by:

- Clinically tooth remained symptom-free in place through-out the follow-up period with lack of pain, swelling, abscess, fistula, and mobility.
- Radiographically lack of bifurcation involvement, periapical radiolucency and pathological root resorption.
- Caries was arrested with no further progression clinically and radiographically.
- Restoration appeared satisfactory with no further intervention required.

Secondary outcomes were:

- Treatment failure were categorised into minor and major failure;
  o Minor failure: if the initial treatment provided has failed, where the tooth remained restorable in place and did not result in the tooth being extracted. This was measured by development of secondary caries, caries progression, restoration loss, and occurrence of reversible pulpitis (clinically pain on eating/drinking lasts for a few seconds) which could be managed by repair or replacement of the restoration.
  o Major failure: if the initial treatment provided has failed, where the tooth inevitably had to be extracted or a pulp treatment had to be performed. This was measured by pulpal exposure during treatment, clinical and radiographic signs or symptoms of irreversible pulpal damage such as dental abscess, fistula, spontaneous pain, periapical radiolucency, bifurcation involvement, or if the tooth is broken down and unrestorable.
- Time to treatment/restoration failure/retreatment measured by months.
- Discomfort associated with the procedure reported at time of the dental appointment or within 24 h of treatment.
- Patient/carer perceptions and acceptance of treatment measured quantitatively or qualitatively.
- Oral health-related quality of life measured quantitatively or qualitatively.
- Adverse events and side effects.

We included systematic reviews (SRs) with and without meta-analyses. We excluded studies reviews in which caries removal was assisted by chemomechanical agents, or procedures were local anaesthesia were used to perform the treatment. We also excluded reviews concentrating on prevention or management of enamel lesions. In addition, reviews presenting pooled data reporting randomised clinical trials (RCTs) and SRs were excluded. For reviews investigating other interventions alongside MID, only MID data were considered.

### Search strategy

An experienced information specialist searched MEDLINE, Embase, Cochrane Database of Systematic Reviews, Epistemonikos, Joanna Briggs Institute Database of Systematic Reviews and Implementation Reports and The NIHR Journals Library. In addition, the PROSPERO database was searched to identify forthcoming SRs.

Searches were built around the following four concepts: primary teeth AND caries/carious lesion AND Minimal Intervention Dentistry AND systematic review/meta-analysis. Searches were restricted to English language SRs and/or meta-analyses published between January 2000 and August 2020. The full search strategies can be found in the Supplementary File (File 1). Reference lists of the included studies were also screened. Search results were downloaded into a reference management software (Endnote, Version 9) and duplicates removed.

### Reviews selection process

Two reviewers (ABH, SH) independently screened all titles and abstracts. Following this, the same investigators screened the full text of studies assessed as being relevant or potentially relevant from the title and abstract screen. Two different reviewers resolved disagreements (RS, SA). Data from all included studies were extracted and assessed using designed data extraction forms.

For each review, the following data were recorded systematically: publication details (authors, year of publication, country of origin, and source of study funding), sample characteristics (participants age, inclusion and exclusion criteria), review methodology (search strategy, PICO items, objectives, number of included studies, study design, sample size, risk of bias assessment tool used, and method of grading the quality of evidence), teeth and any intervention carried out (tooth type, caries depth, surface(s) affected, presence of preoperative radiographs, pulpal condition, intervention and control used including type and method of restoration, extent of carious removal, method of caries removal and follow-up duration), outcome information including methods of assessment and information regarding risk of bias.

### Risk of bias assessment

Risk of bias was conducted independently by two reviewers using the Risk of Bias in Systematic reviews (ROBIS) tool to assess each SRs across three areas:

- Relevance of the review
- Identification of concerns within the SR process under four domains: study eligibility criteria, identification and selection of studies, data collection and study appraisal, and synthesis and findings.
- Judging risk of bias (low, high or unclear risk of bias score). Scoring discrepancies was resolved through discussion until consensus was reached.

### Data synthesis and analysis

The effect estimates (95% confidence intervals), and measures of heterogeneity was analysed for every review. Treatment outcomes (success, minor and major failures) were summarized narratively through tables and synthesis of similar outcome measures were carried out to compare these across comparator interventions.

To determine the overlap in studies across the SRs, citation matrices were generated, and “Corrected Covered Areas” (CCAs) were calculated as follows: CCA = 0–5; slight, 6–10; moderate, 11–15; high, and > 15; very high overlap.

SRs of different types of MID were analysed separately because of the different treatment techniques used in the MID; however, similar populations, interventions, or outcome measures were grouped in this umbrella review.

## Results

### Selection of studies

**Fig. 1:**
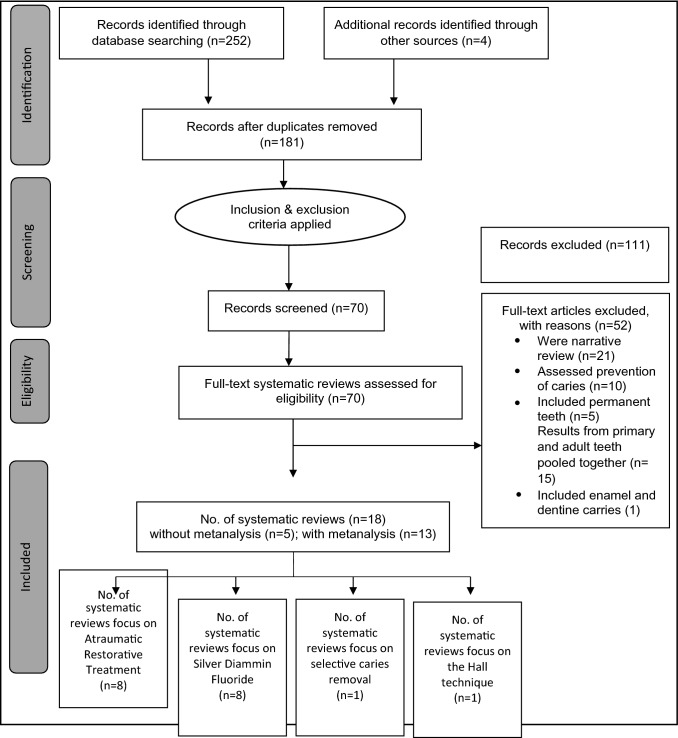
Flow diagram: identification and study selection

The initial search in databases and other sources resulted in 252 records, of which 177 remained after duplicates were removed, and four additional publications added from screening bibliographies resulting in 181 potentially eligible reviews. 162 papers were excluded after title and abstract screening, which left 19 publications eligible for full-text review. One SR was excluded following full text review and 18 SRs, 5 without meta-analysis and 13 with meta-analysis were included, reporting on 95 studies in total. Figure 1 shows the search and assessment process flowchart of the literature search.

### Characteristic of the SRs

From the 18 included SRs, 8 assessed the caries arresting effects of 38% SDF on dentine carious lesions (Duangthip et al. 2015, 2016; Gao et al. 2016; Chibinski et al. 2017; Contreras et al. 2017; Tolba et al. 2019; Jabin et al. 2020), 1 on the HT (Innes et al. 2015), 8 investigated interventions using ART (van’t Hof et al. 2006; Mickenautsch and Yengopal 2012; Raggio et al. 2013; Dorri et al. 2017; Tedesco et al. 2017; de Amorim et al. 2018; Tedesco et al. 2018; Ortiz-Ruiz et al. 2020), and 1 SR assessed selective caries removal technique (Ricketts et al. 2013). Included SRs were published between 2006 and 2020, covering a defined time frame of included RCTs ranging from 1969 to 2018.

Ten systematic reviews used Cochrane risk of bias assessment tool (Ricketts et al. 2013; Duangthip et al. 2015; Innes et al. 2015; Duangthip et al. 2016; Chibinski et al. 2017; Contreras et al. 2017; Dorri et al. 2017; Trieu et al. 2019; de Amorim et al. 2018; Tedesco et al. 2017), four used the Cochrane collaboration common scheme for bias (Mickenautsch and Yengopal 2012; Gao et al. 2016; Tedesco et al. 2018; Tolba et al. 2019), and two used other tools for assessment (Raggio et al. 2013; Jabin et al. 2020). However, two systematic reviews did not use any well-defined assessment tool (van’t Hof et al. 2006; Ortiz-Ruiz et al. 2020) (Table 1).

**Table 1:** Risk of bias according to ROBIS and assessment used in systematic review

Author year	ROBIS Risk of bias	Assessment used
Silver diamine fluoride		
Duangthip (2015)	High Risk	Cochrane risk of bias assessment tool
Duangthip (2016)	High risk	Cochrane risk of bias assessment tool
Gao (2016)	Unclear risk	Cochrane collaboration common scheme for bias
Contreras (2017)	High risk	Cochrane risk of bias assessment tool
Chibinski (2017)	High risk	Cochrane risk of bias assessment tool
Trieu (2019)	Low risk	Cochrane risk of bias assessment tool
Talbo (2019)	Low risk	Cochrane collaboration common scheme for bias
Jabin (2020)	High risk	Oxford centre for evidence-based medicine critical appraisal worksheet for controlled clinical trials
Atraumatic restorative technique		
Van’t Hof (2006)	High risk	No risk of bias conducted
Michenautsch (2012)	High risk	Cochrane collaboration common scheme for bias
Raggio (2013)	High risk	Quality score criteria for therapy articles
Dorri (2017)	Low risk	Cochrane risk of bias assessment tool
Tedesco (2017)	Low risk	Cochrane risk of bias assessment tool
Tedesco (2018)	Unclear risk	Cochrane collaboration common scheme for bias
de Amorim (2018)	High risk	Cochrane risk of bias assessment tool
Ortiz-Ruiz (2020)	High risk	Not mentioned, a funnel plot was used to evaluate publication bias
Hall technique		
Innes (2015)	Low risk	Cochrane risk of bias assessment tool
Selective caries removal		
Rickets (2013)	Low risk	Cochrane risk of bias assessment tool

Table 2 shows the main characteristics of the included SRs according to the considered technique.

**Table 2:** Summary of included systematic reviews characteristics

Author	Year of publication	Types of studies	Number of studies	Range of publication	Meta-analysis conducted	Grading tool used	Funding Source
Systematic reviews on silver diamine fluoride							
Duangthip et al	2015	RCTs	2	2002–2012	No	Cochrane risk of bias assessment tool	Faculty of Dentistry, University of Hong Kong
Duangthip et al	2016	RCTs	1	2012	No	Cochrane risk of bias assessment tool	None stated
Gao et al	2016	RCTs & CRTs	12	1969–2012	Yes	Cochrane collaboration common scheme for bias	None stated
Contreras et al	2017	RCTs	3	2005–2012	No	Cochrane risk of bias assessment tool	National Institutes of Health Award No. HCTRECD R25MD007607 and HiREC S21MD001830 from the National Institute on Minority Health and Health Disparities
Chibinski et al	2017	RCTs	5	2002–2013	Yes	Cochrane risk of bias assessment tool	National Council for Scientific and Technological Development (CNPq) under grants 304,105/2013–9 and 305,588/2014–1
Trieu et al	2019	RCTs	3	2001–2016	Yes	Cochrane risk of bias assessment tool	School of Dental Medicine, University of Nevada, USA
Tolba et al	2019	RCTs	3	2009–2018	No	Cochrane collaboration common scheme for bias	None stated
Jabin et al	2020	RCTs & CRTs	4	2002–2018	No	Oxford Centre for Evidence-based Medicine critical appraisal worksheet for controlled clinical trials	None stated
Systematic Reviews on Atraumatic Restorative Treatment							
van ‘t Hof et al	2006	Not stated	9	1999–2004	Yes	No risk of bias conducted	None stated
Mickenautsch et al	2010	RCTs and CRTs	3	2002–2004	Yes	Cochrane collaboration common scheme for bias	None stated
Raggio et al	2013	RCTs	3	2002–2006	Yes	Quality score criteria for therapy articles	None stated
Dorri et al	2017	RCTs	6	2003–2007	Yes	Cochrane risk of bias assessment tool	The University of Manchester, Manchester Academic Health Sciences Centre (MAHSC), UK NIHR Manchester Biomedical Research Centre, UK
Tedesco et al	2017	RCTs	4	2002–2014	Yes	Cochrane risk of bias assessment tool	None stated
Tedesco et al	2018	RCTs and observatory studies	15	2002–2016	Yes	Cochrane collaboration common scheme for bias	None stated
de Amorim et al. (2018)	2018	RCTs	12	Not stated	Yes	Cochrane risk of bias assessment tool	National Council for Scientific and Technological Development from the Brazilian Government, under grants 309,521/2015–7 and 306,852/2016–0
Ortiz-Ruiz et al. (2020)	2020	RCTs and CRTs	4	2001–2010	Yes	Not mentioned, A funnel plot was used to evaluate publication bias	None stated
Systematic reviews on Hall technique							
Innes et al	2015	RCTs	2	2011–2014	Yes	Cochrane risk of bias assessment tool	School of Dentistry, The University of Manchester, UK Cochrane Oral Health Group Global Alliance, Other National Institute for Health Research (NIHR), UK
Systematic reviews on selective caries removal							
Rickets et al	2013	RCTs and	4	1977–2009	Yes	Cochrane risk of bias assessment tool	University of Dundee Dental School, UK Guy’s, King’s and St Thomas Dental School, UK. Cochrane Oral Health Group Global Alliance, UK. National Institute for Health Research (NIHR), UK

### No dentine carious tissue removal

#### Silver diamine fluoride (38%) for managing dentine carious lesions

The eight included SRs summarised data from 33 RCT and control clinical trials (CCT). Three SRs conducted meta-analysis to synthesise the findings (Gao et al. 2016; Chibinski et al. 2017; Trieu et al. 2019), and five did not include a meta-analysis (Duangthip et al. 2015, 2016; Contreras et al. 2017; Tolba et al. 2019; Jabin et al. 2020). Main outcome measure assessed in these SRs was caries arrest.

There was weak overlap between the reviews using CCA (Table 3). However, when looking into more details three reviews only included primary studies that already have been analysed in previous reviews (Duangthip et al. 2015, 2016; Contreras et al. 2017).

**Table 3:** Primary included studies included in the systematic reviews for dentine carious lesions management in primary teeth

Primary included studies	Systematic reviews on silver diamine fluoride*							
	Duangthip (2015)	Duangthip (2016)	Gao (2016)	Contreras (2017)	Chibinski (2017)	Trieu (2019)	Tolba (2019)	Jabin (2020)
Yoshida (1976)			X					
Tsutsumi (1981)			X					
Wang (1984)			X					
Ye (1995)			X					
Fukumoto (1997)			X					
Lo (2001)						X		
Chu (2002)	X		X		X	X		X
Llodra (2005)			X	X	X			X
Wong (2005)						X		
Huang (2006)			X					
Yee (2009)			X	X	X		X	X
Wong (2011)						X		
Zhi (2012)	X	X	X	X	X			
Seberol (2013)					X			
Fung (2016)							X	
Fung (2018)							X	X

Primary included studies	Systematic reviews on atraumatic restorative treatment**							
	Van’t Hof (2006)	Michenautsch (2012)	Raggio (2013)	Dorri (2017)	Tedesco (2017)	Tedesco (2018)	de Amorim (2018)	Ortiz-Ruiz (2020)
Lo (2001)	X						X	X
Yee (2001)	X							
Taifour (2002)	X	X	X		X	X	X	
Louw (2002)	X					X	X	
Lin (2003)				X				
Honkala (2003)	X	X	X		X	X	X	
Schriks (2003)				X				
Ersin (2006)			X		X	X		
Van den Dungen (2004)				X		X		
Yu (2004)	X	X		X		X	X	
De Menezes (2006)				X			X	
Roeleveld (2006)				X				
Ersin (2006)							X	X
Van Gemert-Schriks (2007)							X	
Ersin (2008)								X
Yassen (2009)							X	
Carvalho (2010)								X
Mijan (2014)						X		
Deepa (2010)							X	
Hilgert (2014)					X			
Molina (2017)							X	
Hilgert (2017)							X	

Primary included studies	Systematic reviews on the Hall technique	
	Ricketts (2013)	Innes (2015)
Innes (2007)	X	
Santamaria (2014)		X
Innes (2011)		X

Primary included studies	Systematic reviews on selective caries removal
	Ricketts (2013)
Magnusson (1977)	X
Ribeiro (1999)	X
Lula (2009)	X
Ohran (2010)	X

*Overlap calculation for SDF SRs:

Overlap = number of articles that reviewed by more than one reviewer/total number of articles = 5/16 = 0.3125

CA (Covered area) = total number of reviewing/number of articles*number of reviewer = 4 + 3 + 4 + 5 + 3 + 10 + 1 + 2/16*8 = 32/128 = 0.25

CCA (Corrected cover area) = (32–16)/(128–16) = 0.143 slight

**Overlap calculation for ART SRs:

Overlap = number of articles that reviewed by more than one reviewer/total number of articles = 9/22 = 0.41

CA (Covered area) = total number of reviewing / number of articles*number of reviewer = 6 + 3 + 3 + 6 + 4 + 7 + 12 + 4/22*8 = 45/176 = 0.256

CCA (Corrected cover area) = (45–22)/(176–22) = 0.15 slight

One SR included coronal caries in primary upper anterior teeth only (Tolba et al. 2019), another one included carious primary anterior teeth and primary molars (Duangthip et al. 2016), and five SRs included data on primary teeth, however, the type of teeth (anterior or molar) was not specified (Duangthip et al. 2015; Gao et al. 2016; Chibinski et al. 2017; Contreras et al. 2017; Trieu et al. 2019; Jabin et al. 2020). In addition, none of the included SRs reported on the depth of caries or the number of tooth surfaces affected included apart from Jabin et al (2020) study which stated the 38% SDF was applied on single and multi-surfaces carious lesions. Age of children in these studies ranged between 2 and 15 years, however, one SR did not state the age range (Gao et al. 2016). Follow-up period ranged between 6 and 48 months.

The SRs included studies comparing 38% SDF to no treatment, placebo or other interventions including other SDF concentrations (12%), GIC restoration, sealants, ART or 5% NaF fluoride varnish application.

Results from included SRs showed that 38% SDF has statistically significant caries arrest effect in children and that its application is more effective than other preventive management strategies including 5% NaF fluoride varnish and sealing with GIC for arresting dentinal caries in the primary dentition (*p* < 0.5).

Caries arrest rate after single application of 38% SDF ranged between 31 and 79%, whereas biannual application has significantly increased the carries arrest rate to 53 and 91%. In addition, the mean number of arrested caries surfaces was significantly higher when 38% SDF was applied (2.5–4.5) compared to comparators (1.3–1.8) (Duangthip et al. 2015; Jabin et al. 2020). Caries arrest rate of 38% SDF was significantly higher than other SDF concentrations (12%) (*p* < 0.001) (Tolba et al. 2019; Jabin et al. 2020) and caries excavation prior to its application did not significantly affect the caries arrest rate (Duangthip et al. 2015). However, one SR reported that excavation of soft dentine prior to SDF application resulted in shorter caries arrest time (Trieu et al. 2019). For the comparators the effectiveness were 41% for 5% NAF fluoride varnish, 82% for GIC, and 15–34% for no treatment.

One meta-analysis reported an overall proportion of caries arrest for 38% SDF was 81% (*p* < 0.001) (Gao et al. 2016). Another meta-analysis reported an odds ratio of 2.44–3.63 favouring SDF for caries arrest (Trieu et al. 2019).

No significant adverse effects were reported from the use of 38% SDF in children other than black staining potential of SDF on carious lesions. One SR assessed carer satisfaction with their child’s dental appearance after the application of 38% SDF compared to comparators including placebo as well as 5% NAF, and found no significant change in the results across the intervention and control group (Trieu et al. 2019). In addition, one study reported the development of reversible, small, white lesions in the oral mucosa (0.6%) (Contreras et al. 2017).

Further details of the included SRs are presented in Tables 4 and 5.

**Table 4:** Study parameters of systematic reviews on silver diamine fluoride

Author	Number of subjects	Age range	Tooth type	Surface affected	Caries depth	Presence of preoperative radiographs	Pulpal condition	Control	Follow-Up
Duangthip et al. (2015)	587	2–5 years	Primary teeth	Not stated	Dentine caries	No	Not stated	No treatment, GIC, 5% NaF	24–30 months
Duangthip et al. (2016)	212	3–4 years	Primary teeth	Not stated	Dentine caries	No	Not stated	No treatment, ART, NAF 5%, saline, GIC sealant	24 months
Gao et al	Not stated	Not stated	Primary teeth	Not stated	Not stated	No	Not stated	No treatment, GIC, 5% NaF	6–48 months
Contreras et al	1640	3–9 years	Primary teeth	Not stated	Not stated	No	Not stated	No treatment, GIC sealant	24–36 months
Chibinski et al	2129	2–15 years	Primary teeth	Not stated	Not stated	No	Not stated	No treatment, ART, NaF vanish, saline	18–36 months
Trieu et al	1054	4 ± 0.8 years	Primary teeth	Not stated	Dentine caries	No	Not stated	Water, 5% NaF	18–30 months
Tolba et al	1864	3–9 years	Upper anterior teeth	Not stated	Dentine caries	No	Not stated	No treatment, SDF 12%	18–30 months
Jabin et al	2691	3–9 years	Primary teeth	Single and multi surfaces	Not stated	No	Not stated	No treatment, 12% SDF, 5% NaF, water	24–36 months

**Table 5:** Results of systematic reviews on silver diamine fluoride

Author	Outcome measure	Main results	Results of meta-analysis	Main conclusion	Adverse events
Duangthip et al (2015)	Caries arrest	Successful -Caries arrest rate at 1 yr; 37% if SDF applied 1 × a year) 53% if SDF applied 2 × a year -Caries arrest rate at 2 years; 79% if SDF applied 1 × a year, 91% if SDF applied 2 × a year -No difference between annual application of GIC and SDF -SDF application 2 × a year increased caries arrest rate -mean number of arrested caries; 2.5 (when caries was excavated and 38% SDF applied), 2.8 (no caries excavation and sdf), 1.5 (NAF with or without excavation 4 × year), 1.3 (no tx) -No difference in caries arrest rate between excavation or no	Not carried out	Limited evidence to support effectiveness of SDF application once/twice a year	Black staining
Duangthip et al. (2016)	Caries arrest	Successful -Caries arrest rates when SDF applied once a year, and twice a year at 24 months review were 79%, 91%, respectively -Effect of annual SDF and GI application on arresting caries did not differ significantly ART success: 39% at 1 year, 3.5% at 2 years; 48% single surface vs 52% multisurface ART caries arrest: 39% at 1 year, 82% at 2 years	Not carried out	Minimally invasive approaches are advantageous in treating dentin caries in primary teeth in preschool	None stated
Gao et al	Caries arrest	Successful -If 38% SDF applied once a year caries arrest rate was; 31–79% -If applied twice a year:85–91%	-Caries arrest rate at 6mo was 86%, at 12mo was 81%, at 18mo was 78%, at 24 mo was 68%, and at > 30mo was 71% -Overall proportion of caries arrest was 81% (95% CI, 68–89%; *p* < 0.001)	38% SDF has statistically significant caries arrest effect on children. Increased application frequency increases caries arrest rate No consensus on its number and frequency of application to arrest caries	Black staining of teeth
Contreras et al	Caries arrest	Caries arrest rate was significantly higher (53%) when 38% SDF was applied twice a year than when 38% SDF (37%) or GIC (28.6) was applied annually (*p* < 0.001) 38% SDF, with or without tea, was significantly more efficient in caries arrest in the primary dentition, at 12 months, than 12% SDF and a control group (*p* < 0.001)	Not carried out	SDF, at 38%, is more effective than other preventive management strategies for arresting dentinal caries in the primary dentition	Black stains; white lesions in oral mucosa in 3 participants
Chibinski et al	Caries arrest	Successful -Mean number of active carious surface at baseline vs number of inactive at follow-up; At 30 months follow-up: 3.82 (SD 0.27) vs 2.49(SD 0.27) following caries excavation and SDF 38% 4.32 (0.34) vs 2.82 (0.3) when 38% SDF was applied with no caries excavation At 36 months: 3.3 (0.3) vs 2.8 (0.3) At 24 months: 7.9 (7.6) vs 6.6 (6.4) with 38% SDF alone and 8.3 (8.5) vs 7.2 (7.6) when SDF 38% was applied with reducing agent	Carried out but all proportions of SDF were included	SDF is more effective than other active treatments or placebo for caries arrest in primary teeth	Black stains were reported in studies using 38% SDF
Trieu et al	Caries arrest	Successful -Statistical significant difference in caries arrest rate by SDF compared to NAF varnish and water -Parental satisfaction with their child’s dental appearance and dental health did not significantly change (*p* < 0.05) -soft tissue removal prior to SDF application did not induce significant difference in caries arrest (cl95%) -38% SDF resulted in a shorter arrest time compared to NAF varnish -Soft tissue excavation resulted in shorter caries arrest time than no excavation	OR for caries arrest = 2.44–3.63 > 1 favouring SDF for the increment of arrested caries rate,-Chu: OR is 2.44 favouring SDF (included 30% SDF in the meta analysis)	SDF is more effective dentine caries arresting than NAF	Black staining
Tolba et al	Caries arrest	Successful -Statistically significant higher mean number of arrested caries surfaces in 38% SDF vs 12% SDF and control at 6,12, 24 months -Caries arrest rate for 38% SDF at 6, 12 and 30 months were 74%, 64%, and 75.7% respectively (*p* < 0.001) -Biannual application increased caries arrest	Not carried out	Caries of primary teeth in children treated with 38% SDF had higher chances of becoming arrested than those treated with 12%SDF. Higher caries arrest if applied biannually rather than annually assessed at 18 and 30 months. If 38% to be applied once a year, its effective but its effectiveness in arresting caries decrease over time	Black staining
Jabin et al	Caries arrest	Successful -38% SDF applied every 12 months and 6 months showed a caries arrest rate of 66.9% and 75.7% -Single application of 38% SDF resulted in (4.5 ± 0.4) or (2.8 ± 0.3) arrested lesions, more than control group (1.8 ± 0.3) -38% SDF is more effective than 12% SDF -There was no difference with/without reducing agent (tannic acid)	Not carried out	38% SDF application can be useful in arresting dental caries	There was a difference of 0.6% between adverse events recorded between test and control groups including nausea, not eating, dysphagia, dyspnea, swelling around lips and face, itchiness, rashes, stomach ache, and diarrhea Examined effects were gingival or mucosal ulceration or swelling and black discoloration

#### Hall Technique for managing dentine carious lesions in primary teeth

Two SRs including three RCTs with meta-analysis comparing the HT to different comparators were included (Ricketts et al. 2013; Innes et al. 2015). However, the SR by Ricketts et al. (2013) aimed to assess different operative caries management techniques in children and adults including selective caries removal, stepwise removal and the HT. With regards to the HT, the SR included one primary RCT (Innes et al. 2007) of which the results were also included in the second SR (Innes et al. 2015) with longer follow-up period, and therefore, this was not reported in details in the current umberlla review. Comparators included non-selective or selective caries removal followed with restoration (GIC, amalgam, composite, conventional preformed metal crowns (PMCs)) or NRCC. Outcome measures were divided into primary outcomes including major failure, reported pain, and satisfaction with dental treatment. Whereas secondary outcomes involved time to restoration failure (retreatment), discomfort associated with procedure, cost, and adverse events. Children ranged in their age between 3 and 10 years. Primary molars with single and multi-surface cavities were included and followed-up for 1–5 years.

Results from this SR regarding the HT as compared to non-selective caries removal to hard dentine showed no significant difference in sign/symptoms of pulpal disease between the two treatment arms. However, a significant reduction in the risk of restoration failure was reported in favour of the HT; 3% in the intervention group compared to 37% in the control group. In addition, more than two-thirds (77%) of the children, and 83% of the carers participated in the primary study preferred HT to non-selective caries removal to hard dentine. In relation to pain as assessed by the dentist, 89% of the children were assessed as experiencing “no pain, discomfort” to “mild, not significant” during the HT intervention, compared to 78% in the control group.

The SR by Innes (2015) did not compare the effectiveness and safety of HT to comparators, however the findings of the study favoured PMCs to conventional restoration particularly when HT was used as they were likely to reduce the risk of major failure or pain as well as discomfort associated at the time of treatment and in the long term. PMCs despite the technique used to place them, were found to cause more gingival bleeding, however, the results were inconclusive (RR 1.09, CI 0.42–2.86).

There was limited data to assess whether conventional PMCs were better than non-restorative caries treatment.

Further details of the included SRs are presented in Tables 6 and 7.

**Table 6:** Study parameters of systematic reviews on Hall Technique

Author	Number of subjects	Age range	Tooth type	Surface affected	Caries depth	Presence of preoperative radiographs	Pulpal condition	Control	Follow-Up
Innes et al	301	3–10 years	Primary molars	Single and multi-surface	Dentine caries l	Yes	Symptomless, with no clinical or radiographic signs of pulpal pathology	Complete or partial caries removal (GIC, amalgam, composite, conventional SSC) and non-restorative caries treatment, no restorations	1–5 years

**Table 7:** Results of systematic reviews on Hall Technique

Author	Outcome measure	Main results	Results of meta-analysis	Main conclusion	Adverse events
Innes et al	-Primary outcomes: major failure, pain, satisfaction with treatment -Secondary outcomes: time to restoration failure/retreatment, discomfort associated with procedure, cost, adverse events	Minor failure -Adverse events; Crowns seemed to cause more gingival bleeding: results were inconclusive (RR 1.09, CI 0.42–2.86) -Results concerning discomfort associated with HT (reported as moderate, intense, very intense during Tx) were inconclusive (RR 1.67, CI 0.65–4.25)	-Results concerning discomfort associated with HT (reported as moderate, intense, very intense during Tx) were inconclusive (RR 1.67, CI 0.65–4.25) -Adverse events; Crowns seemed to cause more gingival bleeding: results were inconclusive (RR 1.09, CI 0.42–2.86) -None of the included studies compared the effectiveness and safety of HT versus conventional restoration	-Findings favoured crowns compared to conventional fillings particularly when HT was used, they are likely to reduce risk of major failure or pain in the long term compared to conventional fillings -HT may reduce discomfort at time of treatment compared to fillings -There was limited data to assess whether crowns are better than non-restorative caries treatment -There was no data to assess the most effective technique for using crowns (conventional vs HT)	Results concerning discomfort associated with HT (reported as moderate, intense, very intense during Tx) were inconclusive (RR 1.67, CI 0.65–4.25)

#### Selective removal of carious tissue

One SR with meta-analysis summarising data from 3 RCTs was included (Ricketts et al. 2013). In this SR, stepwise excavation, and selective caries removal were compared to non-selective caries removal to hard dentine. Single and multiple-surface cavities with caries extending to pulpal half or pulpal quarter of dentine in primary molars were included. However, one study did not specify caries depth. Children ranged in their age between 3 and 11 years. In stepwise excavation and selective removal of caries, one study excavated caries until the operator determined there was a significant risk of pulp exposure with further excavation, while in the other two studies, caries was removed only from enamel–dentine junction along with superficial necrotic dentine from pulpal and axial walls of the cavity. No further attempt was made to remove dentinal caries. Interventions were followed-up for 12 months. Treatment outcomes were divided into primary outcomes including exposure of the dental pulp during caries removal, development of signs/symptoms suggestive of pulpal disease, caries progression, and restoration failure. Whereas secondary outcomes involved oral health related quality of life, patient/carer perceptions of treatment, and patient reported discomfort during treatment.

Data related to stepwise excavation reported no cases of pulp exposure at first entry compared to 14.5% at second entry. Authors reported 69% reduction in risk of pulp exposure in primary molars with stepwise excavation compared to non-selective caries removal to hard dentine (RR 0.31, 95% CI 0.17–0.57, *p* = 0.0002).

With regards to development of signs/symptoms suggestive of pulp necrosis, one study reported one case (1.8%) of pulp necrosis in the stepwise excavation intervention arm compared to three cases (5.5%) in the control arm. However, it was not clear from the publication whether this was a finding at excavation or on review.

Selective caries removal was also found to reduce the risk of pulp exposure by 77% compared to non-selective caries removal to hard dentine in the primary molars (RR 0.24, 95% CI 0.06–0.90; *p* = 0.03) with no difference in pulpal disease between the two treatment arms (RR 0.27, 95% CI 0.05–1.60, *p* = 0.15).

Further details of the included SRs are presented in Tables 8 and 9.

**Table 8:** Study parameters of systematic reviews on selective caries removal

Author	Number of subjects	Age range	Tooth type	Surface affected	Caries depth	Presence of preoperative radiographs	Pulpal condition	Control	Follow-up
Rickets et al	262	3–11 years	Primary molars	Single and multisurfaces	Dentine caries	Yes	Symptomless, vital	Complete caries removal	12 months

**Table 9:** Results of systematic reviews on selective caries removal

Author	Outcome measure	Main results	Results of meta-analysis	Main conclusion	Adverse events
Rickets et al	Primary outcome: exposure of the dental pulp during caries removal, signs/symptoms of pulpal disease, progression of caries, and restoration failure Secondary outcomes: oral health related quality of life, patient/carer perceptions of treatment, and patient discomfort during treatment	Success; For primary teeth, there was a reduction in risk favouring partial caries removal (RR 0.24 (95% CI 0.06–0.90; *p* = 0.03, I2 = 0%) Failure: 3 studies (2 on primary teeth and one included both primary and adult teeth): There was no difference in pulpal disease between partial and complete caries removal (RR 0.27, 95% CI 0.05–1.60, *p* = 0.15, I2 = 0%	Stepwise excavation: for primary teeth, the risk ratio for pulpal exposure during stepwise excavation was RR 0.31 (95% CI 0.17–0.57, *p* = 0.0002, I2 = 0%) and a 69% reduction in risk respectively compared with complete caries removal Partial caries removal: 2 studies combined primary and adult teeth: Overall there was a reduction in risk of exposure of dental pulp favouring partial caries removal (RR 0.23 (95% CI 0.08–0.69, *p* = 0.009, I2 = 0%), a 77% reduction in risk of pulp exposure compared to complete caries removal	Stepwise and partial excavation reduced the incidence of pulp exposure in symptomless, vital, carious primary teeth	None

#### Atraumatic restorative treatment for managing dentine carious lesions

In total, the 8 included SRs on ART reported on 56 clinical trials. The majority of these SRs included RCTs, CCT, and observational studies except one study by Van’t Hof (2006), which did not report on the type of studies included. Meta-analyses were conducted in the eight included SRs to synthesize the findings.

There was weak overlap between the SRs (Table 3). Although one primary study (Honkala et al. 2003) was included in six of the eight reviews, none of the reviews had complete overlap.

Three different outcome measures were assessed in the SRs: survival rate of restoration (van’t Hof et al. 2006; Raggio et al. 2013; Tedesco et al. 2017), caries arrest (de Amorim et al. 2018; Tedesco et al. 2018), and success/failure of restoration (Mickenautsch and Yengopal 2012; Dorri et al. 2017; de Amorim et al. 2018; Tedesco et al. 2018; Ortiz-Ruiz et al. 2020).

Six SRs included carious primary teeth without specifying the type of teeth (Mickenautsch and Yengopal 2012; Raggio et al. 2013; Dorri et al. 2017; de Amorim et al. 2018; Tedesco et al. 2018; Ortiz-Ruiz et al. 2020), while two SRs specified primary molars (van’t Hof et al. 2006; Tedesco et al. 2017). Five SRs included single and multi-surface carious lesions (van’t Hof et al. 2006; Mickenautsch and Yengopal 2012; Dorri et al. 2017; de Amorim et al. 2018; Tedesco et al. 2018), and three SRs included only multi-surface carious lesions (Raggio et al. 2013; Tedesco et al. 2017; Ortiz-Ruiz et al. 2020). Caries depth was only stated in two SRs (Tedesco et al. 2018; Dorri et al. 2017) and defined as lesions extending into dentine. Participants included in the primary studies ranged in their age between 2 and 14 years and were followed-up up to 5 years (range 6 months to 5 years). One study did not report the sample size (Raggio et al. 2013).

Most SRs included studies comparing ART to different conventional restorations including amalgam, conventional GIC, and resin composite (Mickenautsch and Yengopal 2012; Raggio et al. 2013; Dorri et al. 2017; Tedesco et al. 2017). One SR (Tedesco et al. 2018) compared ART to NRCC, HT, and resin sealant. Three SRs did not specify the comparator but presented the original reported data (de Amorim et al. 2018; van’t Hof et al. 2006; Ortiz-Ruiz et al. 2020).

The success rate of single surface ART at 1-year follow-up ranged between 95 and 100%, the latter dropped slightly to 96.7–91% and 86% at 2-and 3-year follow-up, respectively (van’t Hof et al. 2006; Tedesco et al. 2018). The results for multi-surface ART cavities were significantly lower (*p* < 0.0001) where figures ranged between 73 and 100% at 1 year, 52 and 93% at 2 years, and 48.7 and 88% at 3-year follow-up (van’t Hof et al. 2006; Tedesco et al. 2017; Tedesco et al. 2018). Compared to the ART technique, success rate reported for the conventional restorative treatment using different types of restorations including amalgam, resin composite and conventional GIC, ranged between 100–33.6% and 100–42% for single and multi-surface lesions, respectively over 3 years.

With regards to ART restoration failure rate, two SRs reported a significantly higher mean annual failure rate for multi-surface cavities (17%) compared to single surface cavities (4.7–5%) in primary molars over the first 3 years of their placement (van’t Hof et al. 2006; de Amorim et al. 2018).

Three SRs showed that ART restorations had similar survival rates compared to conventional restoration with amalgam and resin composite in single and multi-surface cavities in primary molars (*p* > 0.05) (Mickenautsch and Yengopal 2012; Raggio et al. 2013; Tedesco et al. 2017]. Whereas one SR reported that ART may increase the risk of restoration failure (OR 1.60, CL 1.13–2.27) regardless of the number of surfaces involved compared to conventional restoration with amalgam, resin composite and conventional GIC in the primary dentition over follow-up period of 12–24 months (Dorri et al. 2017).

No significant adverse events associated with ART restoration were reported by any of the included SRs. One SR reported that ART was associated with less discomfort in children aged 6–8 years compared to conventional treatment with local anaesthetic (OR 0.95, CL 0.51–1.79) (Dorri et al. 2017).

Reported ART results varied between SRs, generally due to the diverse criteria for assessing outcomes. However, the SRs showed homogeneously for primary teeth with regard to the mean survival rates of single-surface ART/HVGIC restorations that were significantly higher over 3 years as compared to multiple-surface ART/HVGIC restorations.

Further details of the included SRs are presented in Tables 10 and 11.

**Table 10:** Study parameters of systematic reviews on atraumatic restorative treatment

Author	Number of subjects	Age range	Tooth type	Surface affected	Caries depth	Presence of preoperative radiographs	Pulpal condition	Control	Follow-up
van ‘t Hof et al	Not stated	3 to > 6 years	Primary molars	Single and multi-surface	Not stated	No	Not stated	No control	3 years
Mickenautsch et al	1926 fillings: 1052 ART and 874 Amalgam	5–7 years	Primary teeth	Single and multi surfaces	Not stated	No	Not stated	Amalgam	1–3 years
Raggio et al	1242 fillings: 715 HVGIC, 527 conventional	Not stated	Primary teeth	Multi surface	Not stated	No	Not stated	Amalgam and composite resin	2–3 years
Dorri et al	1107	3–13 years	Primary teeth	Single and multi surfaces	Dentine caries	No	No pulpal involvement	Amalgam, GIC (conventional), and composite resin	2–3 years
Tedesco et al (2017)	1771 fillings: 985 HVGIC, 786 conventional	2–11 years	Primary molars	Multi surface	Not stated	No	Not stated	Amalgam and composite resin	2–3 years
Tedesco et al. (2018)	3,226	2–10 years	Primary teeth	Single and multi-surface	Dentine caries	No	Not stated	Conventional restorative treatment, LVGIC, NaF, IRT, NRCT, ultraconservative treatment, HT, sealing	6 months–5 years
de Amorim et al	Not stated	2–9 years	Primary teeth	Single and multi-surface	Not stated	No	Not stated	No control	1–3 years
Ortiz-Ruiz et al	253 ART fillings	6–14 years	Primary teeth	Multi surface	Not stated	No	Not stated	No control	2 years

**Table 11:** Results of systematic reviews on atraumatic restorative treatment

Author	Outcome measure	Main results	Results of meta-analysis	Main conclusion	Adverse events
Van‘t Hof et al	Survival rate	Success -The mean survival rates of single-surface ART restorations were statistically significantly higher than those of multiple-surface ART restorations in primary teeth after 1 and 2 years (*p* < 0.0001) -The mean annual failure rates of single-surface and multiple-surface ART restorations in primary teeth over the first 3 years was 4.7% and 17%, respectively.-Mean survival rate for single surface HVGIC ART in primary teeth: at 1 year: 95% at 2 year: 91% at 3 years: 86% -Mean survival rate for multisurface HVGIC ART was: at 1 year: 73% at 2 year: 59% at 3 years: 49%	-The meta-analysis showed high mean survival results for single-surface ART restorations in primary dentitions	-High mean survival results for single-surface ART restorations in primary dentitions but that the mean annual failure rate for multiple-surface ART restorations remains rather high	None stated
Mickenautsch et al	Failure rate	**Success** Relative risk of class I restorations between 0.95 and 1.03 for ART vs Amalgam Relative risk of class II restorations between 0.89 and1.12 for ART vs Amalgam There is no statistically significant difference in the success rates of class I ART and amalgam restorations in primary teeth	The relative risks after 12 and 24 months (RR 0.93; 95%CI 0.83–1.06; *p* = 0.26 and RR 1.07; 95%CI 0.91–1.27; *p* = 0.39, respectively) of class I ART and amalgam restorations in primary teeth	No differences could be found in the primary dentition studies over a 2-year follow-up period. ART restorations with high-viscosity GIC appear to be equally successful, and their survival rate may even exceed that of amalgam fillings	None stated
Raggio et al	Survival rate	Success -Success rate of ART varied between 48.7 and 88.9% -Pooled estimate for ART success rate was 1.04	-No difference in survival results between amalgam and ART with high viscosity GIC in occlusoproximal cavities in primary teeth up to 3 year follow up; *p* > 0.05 (odds ratio ranged between 0.298–1.267)	-No difference in survival results between amalgam and ART with high viscosity GIC in occlusoproximal cavities in primary teeth up to 3-year follow-up	None stated
Dorri et al	Failure rate and pain	**Failure** -Restoration failure in the primary dentition between 12 and 36 months: 1.60 (OR) times higher in the ART arm than in the conventional arm (95% CI 1.13–2.27) -Cavity type had no effect on restoration failure (*p* = 0.64) -ART may reduce the pain during procedure compared with control treatment (MD-0.65, 95% CI-1.38–0.07) -The odds of discomfort were reduced with ART in children between six and eight years of age (OR 0.95,95% CI 0.51 to 1.79) compared to conventional treatment with LA	-Compared to conventional treatment using H-GIC, ART may increase the risk of restoration failure in the primary dentition over a follow-up period from 12 to 24 months -Restoration failure: ART GIC vs CT GIC 1.60 [ 1.13, 2.27] for conventional Pain: ART GIC vs CT-0.65 [− 1.38, 0.07]	-Compared to conventional treatment using H-GIC, ART may increase the risk of restoration failure in the primary dentition over a follow-up period from 12 to 24 months -ART may reduce pain during procedure compared with conventional treatment	None stated
Tedesco et al. (2017)	Survival rate	Success -Survival rate of ART: At 1 year: 100%, 83.3%, 80.7% At 2 years: 88.9%, 76.1%, 66.3% At 3 years: 48.7%, 56.4%	-No statistical significant difference between ART and conventional restoration (*p*: 0.06, OR: 1.26. *p*: 0.477, OR: 0.298. *p*: 0.341, OR: 0.710. *p*: 0.055, OR: 0.710)	-ART restorations have similar survival rates compared to conventional treatment and are a viable option to restore occlusoproximal cavities in primary molars	None stated
Tedesco et al. (2018)	Caries arrest and success of restoration	**Success** -success rate of ART in single surface: at 1 years: 100% & 96% at 2 years: 96.7%, 92.3% and 91.4%, at 3 years: 86.1% -Success rate of ART in multisurface: at 1 year: 100% 97.7%, 73%, 88.9% and 83.1% at 2 years: 93.1%, 88.9%, 76.1% and 52% at 3 years: 93.4% and 48.7% at 3.5 years: 88% -Success rate of HT: at 1 year: 98% at 5 years: 92% -Success of UCT in multisurface: 95.7% at 1 year, 88% at 2 years and 3 years -Success rate of NRCT at 1 year was 75% -Success rate of resin sealants: at 1 year was 91.6% and 75% at 1.5 year was 64.5%	-For caries limited to outer third of dentine of occlusal surfaces, resin composite showed higher success rate than sealing with resin sealnat.no difference was found between resin sealants and CT in terms of caries arrest -For all occlusal surfaces, CRT with compomer had highest success rate > ART -For occlusoproximal, HT had highest success rate > NRCT > CTR > ART > UCT -For occlusal and smooth surfaces caries arrest, highest arrest with biannual application of 38%SDF > 3 × N2F > IRT	-Treatment of dentine caries in primary teeth depends on progression depth and surface involved -Few studies exist with high risk of bias to provide evidence to the best treatment option	None stated
de Amorim et al	Caries arrest and failure rate	Failure -The weighted mean annual failure rates for single and multiple surface ART restorations in primary posterior teeth over the first 3 years were 5 and 17%, respectively	Similar to the main results	-Survival of single surface HVGIC ART in posterior primary teeth over the first 3 years is high, whereas for multiple surface over 2 years is at medium level -Compared to the results from previous SR (12 years older), ART showed an effective option for managing caries in primary teeth rather than as an alternative option	None stated
Ortiz-Ruiz et al	Retention, marginal integrity, anatomic form and absence of recurrent caries	**Success** -Retention success rate of ART: 0.93, 0.75, 0.12 -Absence of recurrent caries 0.93, 0.91, 0.72,	Similar to the main results	-Significant relation between cavity form and filling material with worst combination between HVGIC and ART (*p* < 0.001)	None stated

### Study outcome summary of findings

- Interventions involving no dentine carious tissue removal:
  o Regarding fissure sealants and resin infiltration, the evidence available on these techniques were mainly for their sealing ability of enamel caries, and therefore, they are not recommended for the management of dentinal caries lesions in primary teeth
    Two techniques were considered as not involving removal of dentine carious tissue: topical application of 38% SDF and the HT.
  o Regarding SDF, main results showed that 38% SDF applied every 12 months can be advantageous in terms of caries arrest, however biannual application of 38% SDF resulted in higher caries arrest rate vs single application. For 38% SDF, none of the included SRs reported the treatment effectiveness in terms of the depth of the carious lesions.
  o For PMCs, findings favoured these in general compared to conventional restorations particularly when the HT was used. The HT is likely to reduce the risk of major failure or pain in the long-term compared to conventional restorations including amalgam, GIC and resin composite. In addition, it seems that the HT may reduce discomfort at time of treatment compared to the conventional restorations.
  o There was no available data to determine whether PMCs using the HT was better than the topical use of 38% SDF.
- Interventions involving non-restorative caries treatment:
  o Regarding NRCC, the evidence on this technique was very limited, and low in terms of quality. Therefore, no conclusions on this technique can be drawn.
- Interventions involving selective caries removal:
  o Selective and stepwise caries removal seem to reduce the risk of pulp exposure in asymptomatic, vital, carious primary teeth when these techniques were used to treat deep carious lesions (lesions extending into the inner third or quarter of dentine) over CCR.
  o For selective caries removal (one-step), studies that did not re-enter the carious lesions reported no adverse consequences.
  o Comparing selective caries removal (one-step) to stepwise caries, there was limited evidence to determine the superiority of one over the other in terms of pulp symptoms.
  o There is evidence showing that ART using HVGIC may be associated with the risk of restoration failure for multi-surface cavities. However, ART using HVGIC showed to be an adequate management option for treating single-surface carious lesions in primary teeth.

### Assessment of heterogeneity and systematic reviews’ risk of bias

A substantial heterogeneity was found between the final 18 included SRs mainly concerning subject characteristics, depth and extension of treated lesions, restorative materials used, and outcome measures. However, the trend of heterogeneity was similar across all the studies. In addition, databases searched, and reporting criteria considerably differed among these SRs.

Most studies were found to have considerable risk of bias. Ten systematic reviews were at high risk of bias (Van’t Hof et al. 2006; Mickenautsch and Yengopal 2012; Raggio et al. 2013; Duangthip et al. 2015, Duangthip et al. 2016, Chibinski et al. 2017, Contreras et al. 2017, de Amomrim et al. 2018; Jabin et al. 2020; Ortiz-Ruiz et al. 2020), six were at low risk of bias (Ricketts et al. 2013, Innes et al. 2015; Dorri et al. 2017; Tedesco et al. 2017, Trieu et al. 2019; Tolba et al. 2019) and two were at unclear risk of bias (Gao et al. 2016, Tedesco et al. 2018).

**Fig. 2:**
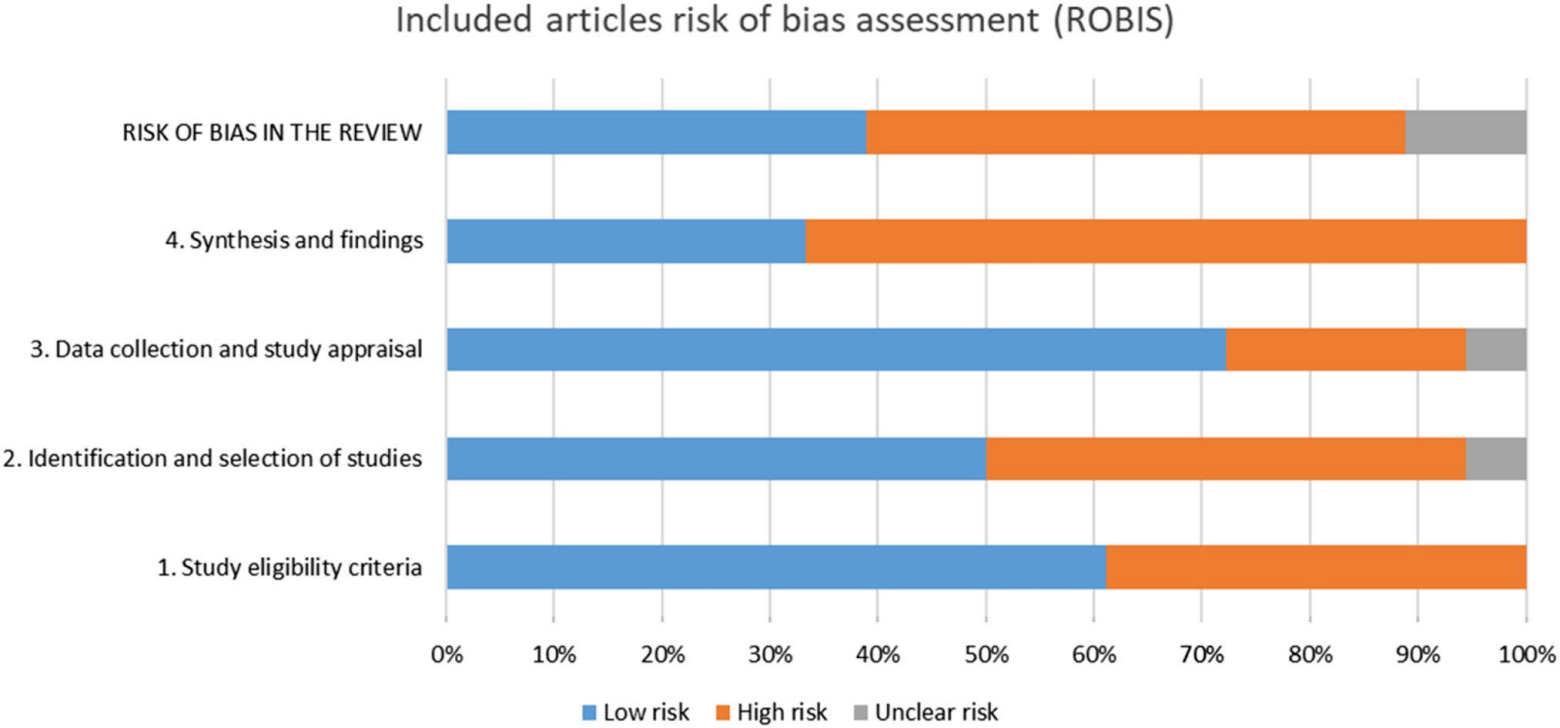
Bias according to ROBIS tool

Figure 2 and Table 1 present a summary of ROBIS assessment across all included reviews.

## Discussion

This umbrella review aimed at investigating the available evidence with regards to the management of carious lesions into dentine in primary teeth using a minimal intervention approach without the need for using local anaesthetic. Due to the availability of many recent SRs, it became clear that an umbrella review approach should be adopted to avoid duplication of available evidence and to comprehensively cover all different techniques that fall under the umbrella of MID. This approach was also supported by the fact that the majority of the published SRs were looking at one modality of MID rather than the full range of techniques incorporated by this term. In total, 18 SRs met the inclusion criteria of the current review and were included in the final review, however, the majority of these reviews focused on 2 main techniques namely 38% SDF (8 SRs included) and ART (8 SRs included). This could be due to the fact that many of the more recently used techniques (e.g., application of SDF) are over taking more established techniques such stepwise and selective caries removal. In addition, we found only one SR looking at the HT (Innes et al. 2015) and another one aimed at assessing the efficacy of ultra-conservative removal of caries which included data on the HT (Tedesco et al. 2018). The latter has also included data on stepwise excavation and selective removal of caries. HT is relatively a new treatment modality which has recently started to have international acceptance. However, there are a couple of ongoing recently registered (PROSPERO; https://www.crd.york.ac.uk/prospero/#searchadvanced) systematic reviews including one is carried out by the authors of the current umbrella review, which will look at the different aspects of the Hall technique, and the results of these ongoing reviews will confidently add to the existing evidence.

As the current umbrella review aimed to investigate the outcomes of MID interventions in the management of dentine rather than enamel caries, SRs assessed the sealing efficacy of fissure sealants and resin infiltration in carious primary teeth were excluded due to pooled data reporting on caries arrest in both enamel and outer third of dentine with the majority of these carious lesions being limited to enamel. Therefore, based on the results of the current review, both fissure sealants and resin infiltration are not recommended for the management of dentinal caries lesions in primary teeth.

The current review provides a broader view of MID in managing dentine carious primary teeth and might be useful to inform guidelines and clinical practice when all of the caries management options need to be contemplated. However, when considering the findings of the current umbrella review, it is important to note that the authors did not analyse the primary studies and therefore the results are subject to the accuracy of the published SRs. In addition, following SR selection, it became clear that it would be difficult to analyse the findings based on the type of caries management intervention (see PICO description), instead each management technique (SDF, HT, selective removal of caries, stepwise and ART) was analysed on its own and the findings of different techniques were brought together.

When included SRs concerning 38% SDF were assessed in the current umbrella review, there was a clearly wide variability in the number of the included primary studies, ranging from 2 up to 12 studies, with some of these studies dated back to 1969. This could be due to the different inclusion criteria adopted in these SRs. However, the main outcome of these reviews was similar (caries arrest). The majority of the included SRs reported a considerable risk of bias in the included studies, and only two SRs reported a low risk of bias even though the included primary studies overlapped (Trieu et al. 2019; Tolba et al. 2019). Independent of the risk of bias, most SRs agreed that a single (annual) application of 38% SDF outperformed the comparators (fluoride varnish, GIC, no treatment or placebo). In addition, the success rate of 38% SDF in arresting dental caries has increased when 38% SDF was applied twice rather than once a year (Duangthip et al. 2016; Gao et al. 2016; Duangthip et al. 2017; Tolba et al. 2019; Jabin et al. 2020). None of the included primary studies compared the topical application of 38% SDF to conventional restorations could explain the fact that this modality of treatment is currently used when conventional restorative techniques are perceived to be challenging to perform. The main drawback reported of SDF application was the potential permanent black staining of carious lesions. One review (Trieu et al. 2019) reported that the latter was not a significant issue for the majority of the included carers in their review.

Looking at the SRs could be identified that authors included studies in which SDF was applied in high-caries-risk children presenting with asymptomatic dentine carious primary teeth with no evidence of pulp damage. These aspects, although often reported in clinical studies, are hardly considered as a variable of analysis and this may influence the long-term success of treatment or might be a factor that should be considered together with the frequency of SDF application.

There is limited evidence from SRs on the HT. The current umbrella review identified only two reviews, both of which included the same primary studies (Ricketts et al. 2013; Innes et al. 2015). In general, our findings revealed that PMCs, favoured successful outcomes (tooth remained symptom-free in place through-out the follow-up time) compared to conventional restorations particularly when the HT was used. In addition, the HT is likely to reduce the risk of major failure (pulp treatment or extraction needed) or pain in the long-term compared to conventional restorations including amalgam, GIC and resin composite. In addition, it seems that the HT may reduce discomfort at time of treatment compared to conventional restorations. There is a clear need for a SR investigating the effectiveness of the HT as well as its cost effectiveness and acceptance by children and parent. An ongoing SR (PROSPERO 2020 CRD42020202442) is taking place in an attempt to answer these questions.

For the selective caries removal, the main limitation to reach conclusions was based on the limited number of included studies with only one systematic review was included in this umbrella review (Ricketts et al. 2013). At the initial search, there was a large number of published reviews on selective caries removal, however, these were not included in the present umbrella review due to substantial heterogeneity of their inclusion criteria (i.e., use of local anaesthesia, pooled data reporting of primary and permanent teeth, the amount of tissue left or removed). This was particularly evident in the reviews which were conducted before the current recommendations on terminology/technique on selective caries removal (consensuses) were published (Schwendicke et al. 2016; Machiulskiene et al. 2020; Splieth et al. 2020a; Santamaría et al. 2020). However, the findings of the current umbrella review showed that particularly in the treatment of deep carious lesions in primary teeth, the use of selective caries removal was beneficial to reduce the risk of pulp exposure. A SR and meta-analysis (Schwendicke et al. 2013) which was not included in present review due to pooled data reporting of primary and permanent teeth has compared selective caries removal (one-step) and stepwise caries removal with non-selective caries removal to hard dentine showed lower risk of pulpal exposure and pulpal symptoms for both management techniques. It is fair to say though the emergence of novel restorative technique with no caries removal such as the HT meant that there is currently less applications for selective caries removal in primary teeth.

With regards to ART, from the 8 SRs included, there was wide variability in the number of included studies ranging from 3 up to 15, with most SRs reporting high risk of bias in the included primary papers. Only two SRs reported a low risk of bias even though the included papers overlapped (Dorri et al. 2017; Tedesco et al. 2017). ART with HVGIC showed to be an adequate management option for treating single-surface carious lesions in primary teeth. However, when ART was used in multi-surface carious lesions, the technique had less success rate with higher mean annual failure rate. It is worth noting that the quality of evidence from these SRs was low and hence the current umbrella review was not able to draw conclusions.

Unlike 38% SDF, ART was compared to conventional restorations in the majority of the reviews. This could be explained by the fact that ART has been considered an alternative treatment option to conventional restoration for carious primary teeth for a long period of time.

Similarly to our findings, a recent systematic review (Santamaría et al. 2020) (not included in the present umbrella review due to pooled data reporting RCTs and SRs) concluded that less invasive caries approaches involving selective or no caries removal and SDF application seem advantageous in comparison with complete caries removal for patients presenting with vital, symptomless, carious dentine lesions in primary teeth.

This umbrella review is not without limitations. Data on different treatment outcomes were reported mainly based on the clinical findings of the included SRs with very limited data were reported from post-operative radiographs. This might have under estimated the rate of the minor and major failures reported on these treatment techniques.

## Conclusion and recommendation

The results of the current umbrella review confirm that despite the heterogeneity of the included SRs and primary studies, MID techniques appear to be effective in arresting the progress of dentinal caries in primary teeth when compared to no treatment and conventional restorations. There is some evidence of improved patient reported outcomes with such techniques, however, further research is required in this area to inform guideline development and to ensure treatment recommendations are based on both clinical outcomes but more importantly child experience and acceptance. There is a clear need to increase the emphasis on utilising MID techniques in managing dentinal caries lesions in primary teeth as a mainstream option rather than a compromise option in circumstances where the conventional approach is prohibited due to cost or cooperation.

## Supplementary Information

Below is the link to the electronic supplementary material.

Supplementary file1 (DOCX 22 KB)
