# Supplementary material for: Minimal intervention dentistry for managing carious lesions into dentine in primary teeth: an umbrella review
Source: Eur Arch Paediatr Dent. 2021 Nov 16;23(5):667–93. doi: 10.1007/s40368-021-00675-6 (PMC9637620; doi:10.1007/s40368-021-00675-6)
Supplement: Supplementary file 1 — Supplementary file1 (DOCX 22 KB) [file 40368_2021_675_MOESM1_ESM.docx]

**Search strategies**

**MEDLINE Ovid**

1. exp Tooth, Deciduous/

2. ((Primary or deciduous or temporary or baby) adj3 (teeth or tooth)).ti,ab,kw.

3. "primary dentition".ti,ab,kw.

4. or/1-3

5. exp Dental Caries/

6. "Carious lesion*".ti,ab,kw.

7. (carious adj (tooth or teeth)).ti,ab,kw.

8. ((tooth or teeth or dental) adj (caries or decay*)).ti,ab,kw.

9. or/5-8

10. 4 and 9

11. exp Dental Atraumatic Restorative Treatment/

12. "minimal intervention".ti,ab,kw.

13. "minimal* invasive".ti,ab,kw.

14. "biological treatment*".ti,ab,kw.

15. "biological approach*".ti,ab,kw.

16. ultraconservative.ti,ab,kw.

17. excavation*.ti,ab,kw.

18. (caries adj2 removal).ti,ab,kw.

19. "atraumatic restorative".ti,ab,kw.

20. (stepwise adj2 removal).ti,ab,kw.

21. (stepwise adj2 excavation).ti,ab,kw.

22. "2-step caries removal".ti,ab,kw.

23. "silver diamine fluoride".ti,ab,kw.

24. "silver diammine fluoride".ti,ab,kw.

25. "diamine silver fluoride".ti,ab,kw.

26. "diammine silver fluoride".ti,ab,kw.

27. "silver fluoride".ti,ab,kw.

28. "Hall crown*".ti,ab,kw.

29. "Hall technique*".ti,ab,kw.

30. "preformed metal crown*".ti,ab,kw.

31. sealing.ti,ab,kw.

32. exp "Pit and Fissure Sealants"/

33. ((fissure or dental) adj seal*).ti,ab,kw.

34. resin infiltration.ti,ab,kw.

35. "ICON system".ti,ab,kw.

36. selective.ti,ab,kw.

37. non-selective.ti,ab,kw.

38. "Non-restorative caries".ti,ab,kw.

39. non-operative caries.ti,ab,kw.

40. (partial adj3 removal).ti,ab,kw.

41. (complete adj2 removal).ti,ab,kw.

42. (incomplete adj3 removal).ti,ab,kw.

43. (partial adj3 excavation).ti,ab,kw.

44. (incomplete adj3 excavation).ti,ab,kw.

45. "indirect pulp".ti,ab,kw.

46. or/11-45

47. 10 and 46

48. limit 47 to english language

49. limit 48 to yr="2000 -Current"

50. limit 49 to (meta analysis or "systematic review")

51. "systematic review".ti,ab,kw.

52. "umbrella review".ti,ab,kw.

53. "meta-analysis".ti,ab,kw.

54. "meta-analyses".ti,ab,kw.

55. "review of reviews".ti,ab,kw.

56. "overview of reviews".ti,ab,kw.

57. 51 or 52 or 53 or 54 or 55 or 56

58. 47 and 57

59. limit 58 to (english language and yr="2000 -Current")

60. 50 or 59

**EMBASE Ovid**

1. exp deciduous tooth/

2. ((Primary or deciduous or temporary or baby) adj3 (teeth or tooth)).ti,ab,kw.

3. "primary dentition".ti,ab,kw.

4. or/1-3

5. exp dental caries/

6. "Carious lesion*".ti,ab,kw.

7. (carious adj (tooth or teeth)).ti,ab,kw.

8. ((tooth or teeth or dental) adj (caries or decay*)).ti,ab,kw.

9. or/5-8

10. 4 and 9

11. exp atraumatic restorative treatment/

12. "minimal intervention".ti,ab,kw.

13. "minimal* invasive".ti,ab,kw.

14. "biological treatment*".ti,ab,kw.

15. "biological approach*".ti,ab,kw.

16. ultraconservative.ti,ab,kw.

17. excavation*.ti,ab,kw.

18. (caries adj2 removal).ti,ab,kw.

19. "atraumatic restorative".ti,ab,kw.

20. (stepwise adj2 removal).ti,ab,kw.

21. (stepwise adj2 excavation).ti,ab,kw.

22. "2-step caries removal".ti,ab,kw.

23. "silver diamine fluoride".ti,ab,kw.

24. "silver diammine fluoride".ti,ab,kw.

25. "diamine silver fluoride".ti,ab,kw.

26. "diammine silver fluoride".ti,ab,kw.

27. "silver fluoride".ti,ab,kw.

28. "Hall crown*".ti,ab,kw.

29. "Hall technique*".ti,ab,kw.

30. "preformed metal crown*".ti,ab,kw.

31. sealing.ti,ab,kw.

32. exp fissure sealant/

33. ((fissure or dental) adj seal*).ti,ab,kw.

34. resin infiltration.ti,ab,kw.

35. "ICON system".ti,ab,kw.

36. selective.ti,ab,kw.

37. non-selective.ti,ab,kw.

38. "Non-restorative caries".ti,ab,kw.

39. non-operative caries.ti,ab,kw.

40. (partial adj3 removal).ti,ab,kw.

41. (complete adj2 removal).ti,ab,kw.

42. (incomplete adj3 removal).ti,ab,kw.

43. (partial adj3 excavation).ti,ab,kw.

44. (incomplete adj3 excavation).ti,ab,kw.

45. "indirect pulp".ti,ab,kw.

46. or/11-45

47. 10 and 46

48. "systematic review".ti,ab,kw.

49. "umbrella review".ti,ab,kw.

50. "meta-analysis".ti,ab,kw.

51. "meta-analyses".ti,ab,kw.

52. "review of reviews".ti,ab,kw.

53. "overview of reviews".ti,ab,kw.

54. or/48-53

55. 47 and 54

56. limit 55 to (english language and yr="2000 -Current")

**Cochrane CDSR and Protocols (The Cochrane Library)**

ID Search Hits

#1 MeSH descriptor: [Tooth, Deciduous] explode all trees

#2 ((Primary or deciduous or temporary) NEAR/3 (teeth or tooth)):ti,ab,kw

#3 ("primary dentition"):ti,ab,kw

#4 {OR #1-#3}

#5 MeSH descriptor: [Dental Caries] explode all trees

#6 (carious NEXT lesion*):ti,ab,kw

#7 (carious NEXT tooth):ti,ab,kw

#8 (carious NEXT teeth):ti,ab,kw

#9 ((tooth or teeth or dental) NEXT (caries or decay*)):ti,ab,kw

#10 {OR #5-#9}

#11 #4 AND #10

#12 MeSH descriptor: [Dental Atraumatic Restorative Treatment] explode all trees

#13 ("minimal intervention"):ti,ab,kw

#14 (minimal* NEXT invasive):ti,ab,kw

#15 (biological NEXT treatment*):ti,ab,kw

#16 (biological NEXT approach*):ti,ab,kw

#17 (ultraconservative):ti,ab,kw

#18 (excavation*):ti,ab,kw

#19 (caries NEAR/2 removal):ti,ab,kw

#20 ("atraumatic restorative"):ti,ab,kw

#21 (stepwise NEAR/2 removal):ti,ab,kw

#22 (stepwise NEAR/2 excavation):ti,ab,kw

#23 ("2-step caries removal"):ti,ab,kw

#24 ("silver diamine fluoride"):ti,ab,kw

#25 ("silver diammine fluoride"):ti,ab,kw

#26 ("diammine silver fluoride"):ti,ab,kw

#27 ("diamine silver fluoride"):ti,ab,kw

#28 ("silver fluoride"):ti,ab,kw

#29 (Hall NEXT crown*):ti,ab,kw

#30 (Hall NEXT technique):ti,ab,kw

#31 (preformed NEXT metal NEXT crown*):ti,ab,kw

#32 (sealing):ti,ab,kw

#33 MeSH descriptor: [Pit and Fissure Sealants] explode all trees

#34 ((fissure or dental) NEXT sealer*):ti,ab,kw

#35 ("resin infiltration"):ti,ab,kw

#36 ("ICON system"):ti,ab,kw

#37 (selective):ti,ab,kw

#38 (non-selective):ti,ab,kw

#39 ("Non-restorative caries"):ti,ab,kw

#40 ("non-operative caries"):ti,ab,kw

#41 (partial NEAR/3 removal):ti,ab,kw

#42 (complete NEAR/2 removal):ti,ab,kw

#43 (incomplete NEAR/3 removal):ti,ab,kw

#44 (partial NEAR/3 excavation):ti,ab,kw

#45 (incomplete NEAR/3 excavation):ti,ab,kw

#46 ("indirect pulp"):ti,ab,kw

#47 {OR #12-#46}

#48 #11 AND #47

**Epistemonikos**

(title:(carious OR caries OR decay*) OR abstract:(carious OR caries OR decay*)) AND (title:((title:("primary teeth" OR "deciduous teeth" OR "baby teeth" OR "temporary teeth" OR "primary tooth" OR "deciduous tooth" OR "baby tooth" OR "temporary tooth") OR abstract:("primary teeth" OR "deciduous teeth" OR "baby teeth" OR "temporary teeth" OR "primary tooth" OR "deciduous tooth" OR "baby tooth" OR "temporary tooth"))) OR abstract:((title:("primary teeth" OR "deciduous teeth" OR "baby teeth" OR "temporary teeth" OR "primary tooth" OR "deciduous tooth" OR "baby tooth" OR "temporary tooth") OR abstract:("primary teeth" OR "deciduous teeth" OR "baby teeth" OR "temporary teeth" OR "primary tooth" OR "deciduous tooth" OR "baby tooth" OR "temporary tooth")))) AND (title:("minimal intervention" OR "minimal* invasive" OR "biological treatment*" OR "biological approach*" OR ultraconservative OR excavation* OR "caries removal" OR "atraumatic restorative" OR stepwise OR 2-step OR "silver diamine fluoride" OR "silver diammine fluoride" OR diamine silver fluoride" OR diammine silver fluoride" OR "silver fluoride" OR "Hall crown*" OR "Hall technique*" OR "preformed metal crown*" OR sealing OR "fissure seal*" OR "dental seal*" OR "resin infiltration" OR "ICON system" OR selective OR non-selective OR "Non-restorative caries" OR "non-operative caries" OR "partial removal" OR "complete removal" OR "incomplete removal" OR "partial excavation" OR "incomplete excavation" OR "indirect pulp") OR abstract:("minimal intervention" OR "minimal* invasive" OR "biological treatment*" OR "biological approach*" OR ultraconservative OR excavation* OR "caries removal" OR "atraumatic restorative" OR stepwise OR 2-step OR "silver diamine fluoride" OR "silver diammine fluoride" OR diamine silver fluoride" OR diammine silver fluoride" OR "silver fluoride" OR "Hall crown*" OR "Hall technique*" OR "preformed metal crown*" OR sealing OR "fissure seal*" OR "dental seal*" OR "resin infiltration" OR "ICON system" OR selective OR non-selective OR "Non-restorative caries" OR "non-operative caries" OR "partial removal" OR "complete removal" OR "incomplete removal" OR "partial excavation" OR "incomplete excavation" OR "indirect pulp"))

**Joanna Briggs Institute (Systematic Reviews)**

(carious OR caries OR decay*) AND ("primary teeth" OR "deciduous teeth" OR "baby teeth" OR "temporary teeth" OR "primary tooth" OR "deciduous tooth" OR "baby tooth" OR "temporary tooth")

**NIHR Journals Library**

carious OR caries OR decay OR decayed Limit to Evidence synthesis

**PROSPERO**

#1 MeSH DESCRIPTOR Tooth, Deciduous EXPLODE ALL TREES

#2 (Primary or deciduous or temporary) adj3 (teeth or tooth)

#3 "primary dentition"

#4 #1 OR #2 OR #3

#5 MeSH DESCRIPTOR Dental Caries EXPLODE ALL TREES

#6 "Carious lesion*"

#7 (carious adj (tooth or teeth))

#8 ((tooth or teeth or dental) adj (caries or decay*))

#9 #5 OR #6 OR #7 OR #8

#10 #4 AND #9

#11 MeSH DESCRIPTOR Dental Atraumatic Restorative Treatment EXPLODE ALL TREES

#12 "minimal intervention"

#13 "minimal* invasive"

#14 "biological treatment*"

#15 "biological approach*"

#16 ultraconservative

#17 excavation*

#18 caries adj2 removal

#19 "atraumatic restorative"

#20 stepwise adj2 removal

#21 stepwise adj2 excavation

#22 "2-step caries removal"

#23 "silver diamine fluoride"

#24 "silver diammine fluoride"

#25 "diamine silver fluoride"

#26 "diammine silver fluoride"

#27 "silver fluoride"

#28 "Hall crown*"

#29 "Hall technique*"

#30 "preformed metal crown*"

#31 sealing

#32 ((fissure or dental) adj sealer*)

#33 resin infiltration

#34 "ICON system"

#35 selective

#36 non-selective

#37 "Non-restorative caries"

#38 non-operative caries

#39 (partial adj3 removal)

#40 complete adj2 removal

#41 incomplete adj3 removal

#42 partial adj3 excavation

#43 incomplete adj3 excavation

#44 "indirect pulp"

#45 MeSH DESCRIPTOR Pit and Fissure Sealants EXPLODE ALL TREES

#46 #11 OR #12 OR #13 OR #14 OR #15 OR #16 OR #17 OR #18 OR #19 OR #20 OR #21 OR #22 OR #23 OR #24 OR #25 OR #26 OR #27 OR #28 OR #29 OR #30 OR #31 OR #32 OR #33 OR #34 OR #35 OR #36 OR #37 OR #38 OR #39 OR #40 OR #41 OR #42 OR #43 OR #44 OR #45

#47 #10 AND #46
